# Examining the role of social determinants of health in maternal mental health screening and treatment engagement during the perinatal period

**DOI:** 10.1186/s13293-025-00687-7

**Published:** 2025-02-12

**Authors:** Leah A. Holcomb, Lizmarie Maldonado, Paul J. Nietert, Marie A. Hayes, Sara M. Witcraft, Roger B. Newman, Kathleen T. Brady, Aimee L. McRae-Clark, Kevin M. Gray, Constance Guille

**Affiliations:** 1 Department of Psychiatry and Behavioral Sciences, Medical University of South Carolina, Charleston, SC USA; 2 Department of Healthcare Leadership and Management, Medical University of South Carolina, Charleston, SC USA; 3 Department of Public Health Sciences, Medical University of South Carolina, Charleston, SC USA; 4 Department of Obstetrics and Gynecology, Medical University of South Carolina, Charleston, SC USA; 5 Department of Psychiatry, Ralph H. Johnson VA Medical Center, Charleston, United States

**Keywords:** Social determinants of health, Substance use, Mental health, Pregnancy, Postpartum

## Abstract

**Background:**

Maternal mental health conditions are associated with unmet Social Determinants of Health (SDOH) needs and can impede access to mental health and substance use disorder (SUD) treatment, leading to poor maternal and newborn health outcomes. A text/phone-based maternal mental health screening and referral to treatment intervention, Listening to Women and Pregnant and Postpartum People (LTWP), has demonstrated improved rates of screening, screening positive for mental health concerns, referral to and attendance of mental health and SUD treatment compared to usual care (i.e., in-person screening and referral). It is unknown, however, if LTWP improves identification of individuals with unmet SDOH needs. This study examines rates of screening, screening positive, referral and attendance to mental health treatment among those with unmet SDOH needs compared to those not experiencing unmet SDOH needs.

**Methods:**

This secondary analysis includes participants randomized to LTWP and endorsing one or more unmet SDOH need (n = 78) or no unmet SDOH need (n = 103) measured by the Accountable Health Communities Health-Related Social Needs Screening Tool via an online survey. Differences in groups' rates of completing a screening, screening positive, being referred to treatment and attending treatment were compared between groups using chi-square tests and relative risk as a measure of association. Adjustments for missing SDOH data via multiple imputations were performed for analysis of the full cohort of LTWP endorsing at least one unmet SDOH need (n = 106) or no unmet SDOH need (n = 118).

**Results:**

Among LTWP participants, 43.0% (78/181) reported at least one unmet SDOH need with financial strain (55.1% (43/78)), disabilities (34.6% (27/78)), and food insecurity (33.3% (26/78)) being the most frequently reported SDOH. On average, participants with SDOH needs were significantly younger (29.0 vs. 32.0 years), more likely to self-identify as non-Hispanic Black (42.3% vs 13.6%), and report a lower household annual income (33.3% vs 1.9% under $25,000), compared to those without SDOH needs. Those with SDOH needs were more likely to screen positive for mental health concerns (RR: 1.59; 95% CI: 1.21–2.09), be referred to (RR: 2.97; 95% CI: 1.36–6.48), and attend mental health treatment (RR: 2.64; 95% CI 1.04–2.73) compared to those without SDOH needs.

**Conclusions:**

The LTWP intervention, a simple text- and phone-based screening approach with referral to care as needed, shows promise in increasing access to mental health and substance use treatment for individuals with unmet social determinants of health needs and demonstrates potential to enhance screening, identification, and treatment attendance rates for perinatal mental health disorders and substance use disorders compared to traditional in-person systems.

**Supplementary Information:**

The online version contains supplementary material available at 10.1186/s13293-025-00687-7.

## Plain Language Summary

Mental health and substance use disorders are commonly experienced by pregnant and postpartum people and can be deadly by suicide or drug overdose. Early identification and treatment can help reduce the adverse effects of these diseases; however, accessing treatment can be challenging, especially when individuals are without financial and social resources (e.g., food, housing, transportation), also known as Social Determinants of Health (SDOH). SDOH, the conditions in which people live and work, can increase the risk of mental health conditions among pregnant and postpartum people and make it more difficult to access treatment. This study examined a text/phone-based screening program, Listening to Women and Pregnant and Postpartum People (LTWP), to determine if the intervention can help identify mental health conditions and facilitate attendance to treatment among those with one or more SDOH needs, compared to those without SDOH needs. In a group of LTWP participants, 43% reported at least one SDOH need. Participants with at least one or more SDOH need were more likely to screen positive for mental health concerns and be referred to and attend mental health treatment compared to those without SDOH needs. These findings suggest that the LTWP intervention can improve the identification and facilitation of mental health treatment for individuals facing SDOH needs during pregnancy and postpartum.

**Supplementary Information:**

The online version contains supplementary material available at 10.1186/s13293-025-00687-7.

## Highlights

- The Listening to Women and Pregnant and Postpartum People (LTWP) text/phone-based maternal mental health screening and referral program increased the likelihood of screening positive for mental health concerns, being referred and attending treatment for mental health and substance use disorders.
- Over 46.0% of individuals enrolled in LTWP reported at least one SDOH need, with financial strain, disabilities, and food insecurity being the most common needs.
- Pregnant and postpartum individuals who self-identified as non-Hispanic Black were disproportionately affected by SDOH needs, emphasizing the importance of addressing racial disparities in both structural and social determinant of health among pregnant and postpartum individuals.

**Supplementary Information:**

The online version contains supplementary material available at 10.1186/s13293-025-00687-7.

## Background

Mental health conditions are a leading cause of maternal mortality and are associated with significant maternal and child morbidity [1]. Most maternal deaths and associated morbidity due to maternal health conditions can be prevented or reduced through appropriate screening, identification, care coordination and treatment for mental health and substance use disorders. Nationally, however, only one in eight individuals will be screened for depression and between 15 and 20% of individuals in need of mental health services will receive treatment [2, 3]. The prevalence of maternal mental health conditions and lack of treatment is even more dire among those with unmet Social Determinants of Health (SDOH) needs.

SDOH, or the conditions in which people live and work, are critical in shaping overall health and well-being and access to treatment [4]. Social determinants of health (SDOH) are non-medical factors, such as economic stability, education, healthcare access, neighborhood conditions, and social context, that significantly influence health and well-being [4]. Unmet SDOH needs refer to gaps or deficiencies in essential non-medical factors—such as economic stability, access to quality education, adequate housing, reliable transportation, healthcare access, safe neighborhoods, and supportive social environments—that negatively impact an individual's overall health, well-being, and ability to access or engage with necessary care and resources [4, 5]. Unmet SDOH have been previously associated with poor health outcomes, including increased risk of mental health and substance use disorders, as well as higher rates of maternal mental health and substance use disorders but lower rates of attendance to treatment [5, 6].

Screening for mental health and substance use concerns/disorders and unmet SDOH needs during pregnancy is recommended by several professional organizations, including the American College of Obstetricians and Gynecologists [3], the American Academy of Pediatrics [7] and the American Psychiatric Association [8]. Regular and systematic screening during prenatal care provides an opportunity to identify and address these issues early, which is critical given their potential to exacerbate maternal morbidity and mortality. Despite these recommendations, implementation of screening and referral practices remains inconsistent due to significant barriers [9, 10]. Patient-, provider-, and system-level barriers can inhibit the uptake of evidence-based mental health screening and referrals during and following pregnancy. Providers' lack of time, unfamiliarity with screening tools, and limited information about available treatment and community resources are frequently cited as primary reasons for not adopting evidence-based screening and referral practices [11, 12]. For patients, lack of knowledge about perinatal mental health and substance use disorders, limited resources for unmet SDOH needs, and stigma often create significant barriers to treatment access and engagement [13, 14]. Fear of punitive actions, such as involvement of child protective services or legal consequences, further discourages many pregnant and postpartum individuals with mental health and/or substance use concerns from seeking care. Addressing these barriers is essential, as inadequate screening and referrals contribute to missed opportunities for intervention. Prenatal care, a consistent period of healthcare contact, is an ideal setting for integrating evidence-based screening and referral practices [10]. Implementing universal and standardized screening for both mental health conditions and unmet SDOH needs during prenatal care has the potential to bridge existing gaps in healthcare access and delivery, allowing for earlier identification of high-risk individuals and more timely interventions. Enhanced screening processes, coupled with robust care coordination, can mitigate the cumulative impact of unmet SDOH needs on maternal health outcomes, ensuring that patients receive the comprehensive support they need during this critical period. Furthermore, investing in such interventions aligns with broader public health efforts to reduce maternal mortality, address health disparities, and promote long-term well-being for mothers and their families. By expanding on and prioritizing these efforts, the healthcare system can move closer to achieving equitable and effective care for all pregnant and postpartum individuals [1, 15].

Prior work suggests that simple text/phone-based screening and referral systems can improve maternal mental health screening rates, screening positive, referral to treatment and attendance to treatment for pregnant and postpartum individuals compared to usual care (in-person screening and referral practices) [16]. One such intervention, Listening to Women and Pregnant and Postpartum People (LTWP), delivers screenings for mental health, substance use, and intimate partner violence via text messaging and conducts brief assessments and referrals to treatment and/or resources via phone, if appropriate, by a care coordinator with a master’s degree in clinical social work. A large randomized controlled trial comparing LTWP to usual care (in-person screening for mental health, substance use, intimate partner violence and referral to treatment and/or resources) found that participants assigned to LTWP were three times more likely to be screened compared with those receiving usual are by a healthcare provider. Additionally, among all participants completing a screen, those assigned to LTWP were 3.1 times more likely to screen positive (i.e., responding positively to screening questions that warrant further evaluation), 4.4 times more likely to be referred to treatment, and 5.7 times more likely to attend mental health or substance use disorder treatment compared with those assigned to usual care [16]. However, it is unknown whether individuals with unmet SDOH needs benefit from LTWP compared to those not experiencing unmet SDOH needs. This secondary analysis explored whether individuals endorsing at least one or more unmet SDOH need benefit from the LTWP intervention compared to individuals assigned to LTWP without unmet SDOH needs.

## Methods

### Study design, setting, and recruitment

Potential participants who had not opted out of being contacted for research were identified via the Electronic Health Record by visit type and approached to take part in the study during a routine prenatal care visit. Eligible participants provided informed consent, and were ages 18–41, English speaking, currently pregnant or postpartum and had received perinatal care within an academic health system in the southeastern United States with plans to continue perinatal care within the health system. Individuals were required to have a cell phone with SMS texting capability and received no prior mental health treatment during the index pregnancy or after birth before enrolling in the study.

Interested participants completed an eligibility screen, and those meeting eligibility requirements provided written informed consent and were subsequently randomly assigned with one-to-one allocation to the study intervention or usual care. All participants were sent a link to completed online study assessments to their phone or email based on participant preference to be completed within the next week. The screening and assessment protocol have been describe previously [16]. Assessments included demographics, medical and psychiatric history and self-reported assessments (Edinburgh Postnatal Depression Scale (EPDS) and the Generalized Anxiety disorder (Gad-7) Scale), as well as the Centers for Medicare and Medicaid Services Accountable Health Communities Health-Related Social Needs Screening Tool (AHC-HRSN) [9, 17]. The AHC-HRSN assesses unmet health-related social needs and factors that may impact health outcomes. The screening tool identifies unmet needs across five core domains. We examined four of the five core domains: housing instability, food insecurity, transportation problems, and utility help needs. We also examined the following supplemental domains: financial strain, employment, family and community support, education, and disabilities. Participants received $25 for completing the online study assessment. The study was approved by an Institutional Review Board and registered at ClinicalTrials.gov (NCT04630249). Study methods were previously published [16].

### Study groups

Participants assigned to LTWP and completed the AHC-HRSN were included in the analyses. Responses from the AHC-HRSN were aggregated and dichotomized such that participants who endorsed at least one or more of the unmet SDOH domains listed above were compared to those who did not endorse any unmet SDOH. Our primary outcomes included the rates of completion of the maternal mental health screening questionnaire during prenatal care (“screened”), responding “Yes” to screening questions that warrant further clinical evaluation (“screened positive”), receiving a referral to treatment (“referred”), and attending an appointment with a psychiatric provider (“attended treatment”) among participants completing the AHC-HRSN and assigned to LTWP. Outcomes were determined by extracting data from the EHR, including screen completion, screened positive and referred to treatment. EHR was also reviewed for attendance to treatment, defined as an EHR encounter/appointment with a psychiatric provider within three months after study enrollment.

Participant demographic information collected includes age, race, ethnicity, annual household income, and the rurality of the participant’s residence. Race and ethnicity were categorized as: “Non-Hispanic Black,” “Hispanic,” “Non-Hispanic White,” and “Other.” Annual household income was collected as “Under $25,000 per year” and “$25,000 per year or more”. Rurality was based on the participant’s county of residence and categorized as rural, partially rural, or urban. Participant obstetric characteristics obtained were pregnant or postpartum status, number of pregnancies, number of living children, and number of weeks pregnant or number of months postpartum.

### Statistical analysis

Descriptive statistics were calculated for both groups (Table 1). Differences in demographic, obstetric and SDOH characteristics between groups were assessed using chi-square or Fisher’s exact tests for categorical variables and *t*-tests or Wilcoxon rank sum tests for continuous variables, as appropriate (Table 1). Unadjusted differences in outcomes were assessed using chi-square tests (Table 2). Relative risk estimates and respective 95% confidence intervals reported were obtained using the FREQ procedure within SAS (Table 2). Potential confounders were considered for adjusted analyses if they were factors unrelated to SDOH classification or were not mediators of outcomes. Analyses adjusting for race were performed and yielded similar results (Supplemental Fig. 1).

**Table 1:** AHC-HRSN Respondent Characteristics

Characteristic	LTWP (n = 181)		LTWP with unmet SDOH Need* (n = 78, 43.09%)		LTWP without unmet SDOH Need* (n = 103, 56.91%)		p-value
	N. or median	% or IQR	N. or median	% or IQR	N. or median	% or IQR	
Age, median years (IQR)^1^	31	28.0–34.0	29	27.0–34.0	32	29.0–34.0	0.0384
*Race and ethnicity, no. (%)*^*2*^							
Non-Hispanic Black	47	25.97	33	42.31	14	13.59	< .0001
Hispanic	11	6.08	4	5.13	7	6.8	
Non-Hispanic White	116	64.09	36	46.15	80	77.67	
Other**	7	3.87	5	6.41	2	1.94	
*Annual household income, no. (%)*^*2*^							
Under $25,000 per year	28	15.47	26	33.33	2	1.94	< .0001
$25,000 per year or more	153	84.53	52	66.67	101	98.06	
*Rurality of patient's residence, no. (%)*^*2*^							
Rural	8	4.68	5	6.76	3	3.09	0.5614
Partially rural	131	76.61	56	75.68	75	77.32	
Urban	32	18.71	13	17.57	19	19.59	
*Obstetric characteristics*							
No. of living children, median (IQR)^1^	1	1.0–2.0	1	1.0–2.0	1	0.0–2.0	0.0747
No. of pregnancies, median (IQR)^1^	2	1.0–3.0	2	1.0–3.0	2	1.0–2.0	0.0425
Currently pregnant, no (%)^2^	87	48.07	31	39.74	56	54.37	0.0511
No. of weeks pregnant, median (IQR)^1^	28	20.0–33.0	23	20.0–28.0	28.5	25.5–33.5	0.0036
No. of months postpartum, median (IQR)^1^	2	1.0–4.5	4	2.0–6.0	1	1.0–1.0	0.0101
*Self-reported psychiatric* *diagnoses, no. (%)*^*2*^							
Mood disorder	25	13.81	21	26.92	4	3.88	< .0001
Anxiety disorder	51	28.18	31	39.74	20	19.42	0.0026
Substance use disorder	19	10.5	16	20.51	3	2.91	0.0001
Psychotic disorder	0	0	0	0	0	0	1
None	118	65.19	38	48.72	80	77.67	< .0001
Self-reported EPDS score, median (IQR)^1^	5	2.0–8.0	7	4.0–12.0	4	2.0–7.0	< .0001
Self-reported GAD-7 score, median (IQR)^1^	3	1.0–6.0	5	2.0–9.0	2	0.0–5.5	0.0007
Total AHC-HRSN Items Endorsed, median (IQR)	–	–	2	1.0–4.0	–	–	
*AHC-HRSN Domains (% Endorsed)*							
Housing instability			22	28.21			
Food insecurity			26	33.33			
Transportation problems			10	12.99			
Utility help needs			13	16.67			
Financial strain			43	55.13			
Employment			23	29.87			
Family and community support			21	26.92			
Education			23	29.49			
Disabilities			27	34.62			

AHC-HRSN = Accountable Health Communities Health-Related Social Needs Screening Tool; LTWP = Listening to Women and Pregnant and Postpartum People; SDOH = Social Determinants of Health; IQR = Interquartile Range; EPDS = Edinburgh Postnatal Depression Scale; GAD-7 = General Anxiety Disorder-7

^*^unmet SDOH need defined as having endorsed at least 1 AHC item

^**^Other includes American Indian or Alaska Native, Asian, and Native Hawaiian or Other Pacific Islander

^1^Wilcoxon Mann–Whitney Test

**Table 2:** Comparison of primary and secondary outcomes among participants assigned to LTWP (N = 181)

	LTWP with unmet SDOH Needs (n = 78, 43.09%)		LTWP without unmet SDOH Needs (n = 103, 56.91%)				
Primary (among all LTWP participants with known AHC-HRSN score)	N	%	N	%	Relative Risk	95% CI	p-value
Screened	78	100	103	100			
Screened positive	53	67.95	44	42.72	1.59	1.21, 2.09	0.0008
Referred to treatment	18	23.08	8	7.77	2.97	1.36, 6.48	0.0062
Attended Treatment	12	15.38	6	5.83	2.64	1.04, 6.73	0.0417
Secondary							
Screened positive (among those who were screened)	53	67.95	44	42.72	1.59	1.21, 2.09	0.0008
Referred to treatment (among those who screened positive)	18	33.96	8	18.18	1.87	0.9, 3.88	0.0937
Attended treatment (among those who were referred to treatment)	12	66.67	6	75	0.89	0.53, 1.49	1

LTWP = Listening to Women and Pregnant and Postpartum People; SDOH = Social Determinants of Health; CI = Confidence Interval

**Fig. 1:**
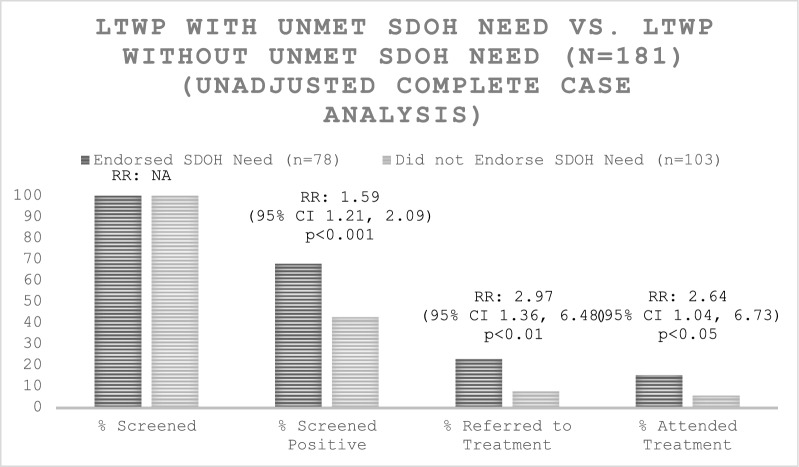
Unadjusted case analysis comparing overall screening, screening positive, referred to treatment, and attendance of treatment for mental health concerns among participants in the LTWP study group with and without SDOH needs

Approximately 18% of the sample did not complete the AHC-HRSN and consequently were missing SDOH information. The multiple imputation by chained equations method was performed on SDOH, with 25 imputation runs, using race and ethnicity, income, patient status (pregnant or postpartum), rurality, number of pregnancies and age as input. That is, known participant demographic characteristics were used to estimate unmet SDOH needs for participants with missing SDOH information. A logistic regression model was used to estimate the overall proportion of SDOH participants, given participants with known unmet SDOH needs and imputed data for participants with unknown SDOH needs. This process was repeated for each outcome as needed to perform analyses. All unadjusted analyses were then repeated using the full cohort of participants with known unmet SDOH needs as well as imputed unmet SDOH needs (Supplemental Table 1). Relative risk estimates and their respective 95% confidence intervals were generated using a log-binomial regression model or a modified Poisson approach with robust error variance to account for zero cells, where appropriate [18]. Results from the imputed data were similar in all but one outcome and are reported in Supplemental Table 1. All hypothesis tests were two-sided, and statistical significance was determined at the 0.05 level. All analyses were performed using SAS statistical software, version 9.4 [19].

## Results

### Participant characteristics

A total of 789 people were screened for study eligibility for the larger randomized controlled trial between January 2021 and April 2023. Of the 575 who were eligible for the study, a total of 415 participants (72.2%) were enrolled and randomly assigned to LTWP (n = 224) or usual care (n = 191). As previously shown, there were no significant differences between the LTWP and usual care groups regarding demographic, obstetric, or mental health history [16]. Of the LTWP participants enrolled, 181 (80.8%) participants completed the AHC-HRSN assessment, and over 43% reported at least one or more unmet SDOH need (Table 1).

With respect to SDOH, financial strain (55.1%) was the most common unmet SDOH need, followed by disabilities (34.6%), food insecurity (33.3%), employment (29.9%), education (29.5%), housing instability (28.2%), family and community support (26.9%), utility help needs (16.7%) and transportation problems (12.9%) among those who endorsed at least one or more SDOH. Responses to AHC-HRSN domain-specific questions can be found in Supplemental Table 2. Supplemental Table 2 provides a detailed breakdown of all LTWP participants who responded to the survey. Among the 181 respondents, 138 (76%) reported no financial need, and over 87% indicated no food insecurity. In contrast, among the 78 participants who reported at least one unmet social determinant of health (SDOH) need, 43 (55%) specifically identified a financial need. The denominators differ between the tables because Supplemental Table 2 focuses on the entire population of LTWP respondents, whereas the analysis in the main text and Table 1 is limited to participants who reported at least one unmet SDOH need. This shift in focus aims to emphasize the specific SDOH domains most frequently endorsed by those with identified unmet SDOH needs.

There were significant differences in demographic, obstetric and mental health variables among those assigned to LTWP and endorsing at least one or more AHC-HRSN items compared to those assigned to LTWP who did not endorse an AHC-HRSN item (Table 1). LTWP participants with one or more unmet SDOH need were significantly younger (29.0 years vs. 32.0 years), self-identify as non-Hispanic Black (42.3% vs. 13.6%), with a greater proportion reported having a lower household annual income (33.3% vs 1.9% below $25,000). In addition, pregnant participants with one or more unmet SDOH need were earlier in pregnancy, compared to those without an unmet need (median of 23 weeks (20.0–28.0) vs 28.5 weeks (25.5–33.5)). Postpartum participants with one or more unmet SDOH need were later in the postpartum period compared to those without an unmet SDOH need [median of four months (2.0–6.0) vs one month (1.0–1.0)]. Individuals who reported at least one unmet SDOH need also self-reported higher rates of mood (26.9% vs. 3.9%), anxiety (39.7% vs. 19.4%), and substance use disorders (20.5% vs. 2.9%) than those without an unmet SDOH need (Table 1).

### Primary outcome

To assess the benefit of the LTWP intervention for those with an unmet SDOH need, LTWP participants endorsing at least one unmet SDOH need were compared to LTWP participants without unmet SDOH needs. All LTWP participants were screened irrespective of SDOH needs. LTWP participants who endorsed an unmet SDOH need were 1.59 (RR, 95% CI 1.21–2.09) times more likely to screen positive, 2.97 (RR, 95% CI 1.36–6.48) times more likely to be referred to treatment, and 2.64 (RR, 95% CI 1.04–6.73) times more likely to attend treatment than participants in LTWP who did not endorse an unmet SDOH need (Fig. 1, Table 2). Analysis adjusting for race demonstrated similar results (Supplemental Fig. 1). Analyses were not adjusted for age as age is considered a SDOH. Analyses were not adjusted for income or number of children as these were correlates of SDOH.

### Secondary outcome

Participants with one or more unmet SDOH need were more likely to screen positive for mental health concerns, and therefore more likely to have the opportunity for referral and attendance to treatment, compared to those without unmet SDOH needs. Thus, we examined group differences in rates of being referred to treatment among those who screened positive and attending treatment among those that were referred to treatment. Differences between groups were not statistically significant (Table 2).

## Discussion

This secondary analysis of a randomized controlled trial comparing Listening to Women and Pregnant and Postpartum People (text/phone-based maternal mental health, substance use, and intimate partner violence screening and referral to treatment program) identified that individuals assigned to the text/phone-based intervention and reporting at least one unmet SDOH need were more likely to screen positive for mental health and/or substance use concerns. The finding that individuals with unmet SDOH needs are more likely to screen positive and, therefore, be referred to treatment is consistent with the current literature that demonstrates higher rates of maternal mental health conditions among those with unmet SDOH needs compared to those without unmet SDOH needs [20, 21]. Although birthing people from all backgrounds are at risk for experiencing maternal mental health problems, low education, low income, and being unemployed all increase the risk of developing postpartum depressive symptoms [22].

Our findings suggest that financial strain is the most common SDOH, consistent with the extant literature. Numerous studies have found that participants with low household incomes report a greater incidence of psychological distress than those with higher incomes [23]. Food insecurity and disabilities were also frequently reported in our sample. Pregnant people living in households with food insecurity have a higher likelihood of experiencing elevated depressive symptoms, emphasizing the importance of early and routine screening for unmet SDOH needs during the prenatal period [24]. Other commonly reported unmet SDOH needs, including lower educational levels, lack of family and community support, housing instability, and transportation issues, all contribute to pregnant people’s experiences of poor mental health symptoms, as well as increase the risk of neonatal and infant health complications [25–27]. The deprivation of these basic and critical daily needs often contributes to pregnant women’s experiences of mental health symptoms, highlighting the importance of adequately screening and connecting individuals to resources to minimize the impact of SDOH disparities. [28]. Given that these risk factors are easily identifiable and often precede the onset of depressive symptoms, early identification of risk and preventative interventions, including access to SDOH resources, should be standard of care [10, 28]. These findings further support the need for policy changes that adequately support the basic needs of pregnant and postpartum people, such as paid maternity leave, subsidized income, food, housing, transportation and childcare.

Of significant concern is that over 40% of the participants recruited from a general prenatal care practice identified at least one unmet SDOH need, and this finding disproportionately impacted non-Hispanic Black individuals. While 42.3% of individuals endorsing more than one unmet SDOH need self-identified as non-Hispanic Black, only 13.5% of those without an unmet SDOH identified as non-Hispanic Black, which underscores the significant unmet needs of our communities and the racial disparities in SDOH. SDOH are a major contributor to the significant racial disparities in maternal morbidity and mortality, with Black birthing persons being two to four times more likely to die compared to White birthing persons [6]. These racial inequities are driven by historical and present-day structural determinants of health, i.e., the social, economic and political factors that create and maintain health inequities and result in individual SDOH disparities [29]. Addressing existing structural determinants of health is necessary to reduce the impact of SDOH and racial disparities in maternal health outcomes.

These results demonstrate that those assigned to LTWP and reporting one or more unmet SDOH need and referred to treatment are more likely to attend mental health treatment. These findings are notable given the lower rates of mental health treatment among those with unmet SDOH needs compared to those not experiencing SDOH disparities [30]. The findings from this study underscore the importance of integrating technology-based interventions into prenatal and postpartum care, particularly for populations disproportionately affected by unmet SDOH needs. A key implication of this study is the potential for text/phone-based screening programs to alleviate the burden on in-office providers, allowing for a more efficient referral process for patients needing SDOH resources and mental health and substance use disorder treatment. The implementation of text/phone-based services, such as those employed in the LTWP intervention, could substantially improve access to care for individuals residing in underserved communities with limited availability of mental healthcare. Providing continuous access to screening and follow-up care outside of traditional healthcare settings offers a promising solution to the geographic and logistical barriers often faced by individuals in remote areas [31]. Additionally, the high attendance to treatment among those with unmet SDOH needs may be explained by care coordinators' unique skillset. LTWP employs a care coordinator with a master’s degree in clinical social work trained to assess mental health and SDOH needs and assist with access to resources that often impede consistent treatment attendance. Including a care coordinator in LTWP appears to be potentially beneficial in promoting treatment attendance among individuals with unmet SDOH needs. However, given that LTWP is a multicomponent intervention, it is unknown if these specifically trained care coordinators are necessary to facilitate attendance to treatment successfully. This raises questions about the specific components of the intervention that drive success, particularly the role of care coordinators with specialized training. Future work is needed to understand the mechanism of action of LTWP and determine if care coordinators with different training and backgrounds are effective for different groups.

### Limitations

Several limitations may affect the interpretation and generalizability of these findings. While results from the imputed data were similar, the results may be biased due to missing SDOH data. Attendance to treatment data were captured in the EHR, and it is possible that participants in either group, regardless if they reported unmet SDOH needs received social services outside of the healthcare system. Therefore, the overall benefit of LTWP for those with unmet SDOH needs, compared to those without, could be magnified. This study occurred in a single healthcare system, and such findings might not be generalizable to other health systems. Furthermore, the study population included only participants who were regularly engaging with the healthcare system to receive prenatal care and who had a cell phone with texting capabilities, potentially limiting generalizability to populations with less consistent healthcare engagement or access to technology. Additionally, our sample was primarily White or African American/Black, and, moreover, there were significantly more non-Hispanic Black individuals in the unmet SDOH need group (42.3%) compared to those who did not endorse unmet SDOH needs (13.6%), and as such the identified unmet SDOH needs might not be reflective of other racial or ethnic groups’ experiences.

### Perspectives and significance

Our findings highlight the significant racial disparities in unmet SDOH needs among participants. Non-Hispanic Black pregnant and postpartum individuals disproportionately (> 40%) represented those with more than one unmet SDOH need, findings reflective of broader structural determinants of health and are deeply rooted in historical and contemporary social, economic, and political systems that maintain racial inequities. The persistence of such disparities is a key contributor to the well-documented racial gaps in maternal morbidity and mortality, with non-Hispanic Black peripartum individuals continuing to experience significantly worse outcomes compared to other racial and ethnic groups [32, 33]. Legislation and resource allocation to address structural determinants of health are necessary to reduce racial disparities in maternal morbidity and mortality. In addition to addressing unmet SDOH, allocation of resources is also necessary to facilitate access to preconception, prenatal and postpartum care, including community-based care through community health workers, doulas, and midwives [31, 35]. Raising awareness about the disparities in maternal health can help drive public support for policy changes and increase community engagement.

The prevalence of unmet SDOH needs overall, and the overrepresentation of unmet SDOH needs among Black individuals in this study is particularly alarming in light of the recent reversal of *Roe v. Wade*. Research indicates that the most frequent demographic group seeking abortion services consists of individuals who already have children and are unable to bear the financial burden of raising an additional child [34]. Seminal findings from the Turnaway Study demonstrated that individuals who sought but were unable to access abortion services experienced significantly worse socioeconomic and health outcomes compared to those who successfully obtained an abortion [35]. Prior studies have shown that financial instability and concerns for existing children are common reasons for seeking abortion services, underscoring the complex interplay between SDOH and reproductive health care access. Interventions such as LTWP, which provide an easy-to-access and confidential disclosure pathway for unmet SDOH needs, can help to reduce these health and racial disparities by improving early and consistent identification and subsequent referral to resources and care for individuals experiencing unmet SDOH needs. However, policy changes are ultimately needed to dismantle harmful structural and social determinants of health and safeguard protections for reproductive rights necessary to achieve health equity.

## Conclusions

The findings from this study emphasize the importance of SDOH screening and referral to care in perinatal care settings, particularly for marginalized populations. The LTWP intervention, which provides a simple text/phone-based screening approach and subsequent referral to care when needed, demonstrates potential to increase access to mental health and substance use treatment among individuals with unmet SDOH needs. It is important to note that these findings are most applicable to populations already engaged in prenatal care. While the LTWP model shows promise in supporting pregnant individuals with unmet SDOH needs within the healthcare system, further research is necessary to evaluate its effectiveness among those who face greater barriers to healthcare access and are less likely to engage in prenatal care. However, by reducing the screening and referral burden on in-office providers, text-message-based models like LTWP can serve as a promising approach to improving access to care for pregnant and postpartum individuals, particularly those facing social and structural barriers.

## Supplementary Information

Supplementary material 1.

Supplementary material 2.

## Data Availability

The datasets used and/or analyzed during the current study are available from the corresponding author on reasonable request.
