# Supplementary material for: Examining the role of social determinants of health in maternal mental health screening and treatment engagement during the perinatal period
Source: Biol Sex Differ. 2025 Feb 12;16:11. doi: 10.1186/s13293-025-00687-7 (PMC11823023; doi:10.1186/s13293-025-00687-7)
Supplement: Supplementary file 1 — Supplementary material 1. [file 13293_2025_687_MOESM1_ESM.docx]

**Figure 2.** Adjusted case analysis, controlling for race, comparing overall screening, screening positive, referred to treatment, and attendance of treatment for mental health concerns among participants in the LTWP study group with and without SDOH needs.
