# Supplementary material for: Examining the role of social determinants of health in maternal mental health screening and treatment engagement during the perinatal period
Source: Biol Sex Differ. 2025 Feb 12;16:11. doi: 10.1186/s13293-025-00687-7 (PMC11823023; doi:10.1186/s13293-025-00687-7)
Supplement: Supplementary file 2 — Supplementary material 2. [file 13293_2025_687_MOESM2_ESM.docx]

| **Supplemental Table 1. Comparison of primary and secondary outcomes among participants assigned to LTWP using imputed data (N=224)** | | | | | | | |
| --- | --- | --- | --- | --- | --- | --- | --- |
|  | **LTWP with unmet**  **SDOH Needs**  **(n=106, 47.32%)** | | **LTWP without unmet SDOH Needs**  **(n=118, 52.68%)** | |  |  |  |
| **Primary (among all LTWP participants with known AHC-HRSN score)** | **N** | **%** | **N** | **%** | **Relative Risk** | **95% CI** | **p-value** |
| Screened | 103 | 97.17 | 117 | 99.15 | 0.97 | 0.94, 1.01 | 0.1472 |
| Screened Positive | 67 | 63.21 | 54 | 45.76 | 1.38 | 1.06, 1.79 | 0.0135 |
| Referred to Treatment | 21 | 19.84 | 10 | 8.47 | 2.36 | 1.09, 5.14 | 0.0302 |
| Attended Treatment | 13 | 12.26 | 7 | 5.93 | 2.06 | 0.81, 5.19 | 0.1274 |
| **Secondary** |  |  |  |  |  |  |  |
| Screened Positive (among those who  were screened) | 67 | 65.05 | 54 | 46.15 | 1.42 | 1.10, 1.83 | 0.0071 |
| Referred to treatment (among those  who screened positive) | 21 | 31.34 | 10 | 18.52 | 1.69 | 0.87, 3.28 | 0.1082 |
| Attended Treatment (among those  who were referred to treatment) | 13 | 61.9 | 7 | 70 | 0.88 | 0.52, 1.50 | 1 |

Note: LTWP = Listening to Women and Pregnant and Postpartum People; SDOH = Social Determinants of Health; CI = Confidence Interval.

| **Supplementary Table 2. AHC-HRSN Question Endorsement (LTWP Participants n=181)** | | | |
| --- | --- | --- | --- |
| **AHC-HRSN Domain** | **Item and Available Responses** | **N Endorsed** | **% Endorsed** |
| Housing Instability | *What is your living situation today?* |  |  |
|  | I do not have a steady place to live (I am temporarily staying with others, in  a hotel, in a shelter, living outside on the street, on a beach, in a car,  abandoned building, bus or train station, or in a park) | 3 | 1.66 |
|  | I have a place to live today, but I am worried about losing it in the future | 6 | 3.31 |
|  | I have a steady place to live | 172 | 95.03 |
|  | *Think about the place you live. Do you have problems with any of the following?* |  |  |
|  | Pests such as bugs, ants, or mice | 12 | 6.63 |
|  | Mold | 3 | 1.66 |
|  | Lead paint or pipes | 0 | 0 |
|  | Lack of heat | 1 | 0.55 |
|  | Oven or stove not working | 0 | 0 |
|  | Smoke detectors missing or not working | 4 | 2.21 |
|  | Water leaks | 4 | 2.21 |
|  | None of the above | 166 | 91.71 |
| Food insecurity | *Within the past 12 months, you worried that your food would run out before you got money to buy more.* |  |  |
|  | Often true | 2 | 1.10 |
|  | Sometimes true | 22 | 12.15 |
|  | Never true | 157 | 86.74 |
|  | *Within the past 12 months, the food you bought just didn't last and you didn't have money to get more.* |  |  |
|  | Often true | 3 | 1.66 |
|  | Sometimes true | 16 | 8.84 |
|  | Never true | 161 | 88.95 |
|  | Missing | 1 | 0.55 |
| Transportation Problems | *In the past 12 months, has lack of reliable transportation kept you from medical appointments, meetings, work or from getting things needed for daily living?* |  |  |
|  | Yes | 10 | 5.52 |
|  | No | 170 | 93.92 |
|  | Missing | 1 | 0.55 |
| Utility help needs | *In the past 12 months, has the electric, gas, oil, or water company threatened to shut off services in your home?* |  |  |
|  | Yes | 13 | 7.18 |
|  | No | 168 | 92.82 |
| Financial strain | *How hard is it for you to pay for the very basics like food, housing, medical care, and heating?* |  |  |
|  | Very hard | 7 | 3.87 |
|  | Somewhat hard | 36 | 19.89 |
|  | Not hard at all | 138 | 76.24 |
| Employment | *Do you want help finding or keeping work or a job?* |  |  |
|  | Yes, help finding work | 19 | 10.50 |
|  | Yes, help keeping work | 4 | 2.21 |
|  | I do not need or want help | 157 | 86.74 |
|  | Missing | 1 | 0.55 |
| Family and Community Support | *If for any reason you need help with day-to-day activities such as bathing, preparing meals, shopping, managing finances, etc., do you get the help you need?* |  |  |
|  | I could use a little more help | 16 | 8.84 |
|  | I get all the help I need | 44 | 24.31 |
|  | I don't need any help | 119 | 65.75 |
|  | Missing | 2 | 1.1 |
|  | How often do you feel lonely or isolated from those around you? |  |  |
|  | Always | 1 | 0.55 |
|  | Often | 5 | 2.76 |
|  | Sometimes | 26 | 14.36 |
|  | Rarely | 61 | 33.7 |
|  | Never | 88 | 48.62 |
| Education | *Do you speak a language other than English at home?* |  |  |
|  | Yes | 11 | 6.08 |
|  | No | 170 | 93.92 |
|  | *Do you want help with school or training? For example, starting or completing job training or getting a high school diploma, GED or equivalent.* |  |  |
|  | Yes | 13 | 7.18 |
|  | No | 168 | 92.82 |
| Disabilities | *Because of a physical, mental, or emotional condition, do you have serious difficulty concentrating, remembering or making decisions?* |  |  |
|  | Yes | 23 | 12.71 |
|  | No | 158 | 87.29 |
|  | *Because of a physical, mental, or emotional condition, do you have difficulty doing errands alone such as visiting a doctor's office or shopping?* |  |  |
|  | Yes | 17 | 9.39 |
|  | No | 163 | 90.06 |
|  | Missing | 1 | 0.55 |

Note: AHC-HRSN = Accountable Health Communities Health-Related Social Needs Screening Tool; LTWP = Listening to Women and Pregnant and Postpartum People; SDOH = Social Determinants of Health.
